# Prevalence of urolithiasis in Sarawak and associated risk factors: An ultrasonagraphy‐based cross‐sectional study

**DOI:** 10.1002/bco2.152

**Published:** 2022-04-20

**Authors:** Kamal Raj Perumal, Richelle Huey Bing Chua, Guan Chou Teh, Clarence Chang Moh Lei

**Affiliations:** ^1^ Department of Urology Sarawak General Hospital Sarawak Malaysia

**Keywords:** prevalence, urolithiasis

## Abstract

**Objective:**

The aim of this research is to study the prevalence of urolithiasis among the population of Sarawak Malaysia and the associated risk factors.

**Patients and Methods:**

A survey was conducted among individuals aged ≥18 years age in three primary health care clinics in the main cities of Sarawak from March 2019 to March 2020. Participants underwent face‐to‐face interview using a predesigned and standardised questionnaire. Details on demographic data, comorbidities, dietary variables and lifestyle were collected. Ultrasonographic examination of the kidney, ureter and bladder was performed followed by blood and urine sampling. Prevalence was defined as the proportion of participants with kidney stones, and univariate logistic regression was used to estimate the associated factors.

**Results:**

A total of 1087 participants (486 male, 601 female) completed the questionnaire. Ultrasonographic examination and laboratory investigation were carried out, with an overall response rate of 98.8%. The prevalence of ultrasonographic proven urolithiasis in the sample studied was 4.04%. The mean age of patients with urolithiasis was 50.05 (SD 14.6, range 18–89), and the male to female ratio was 1.2: 1. Univariate analysis showed that odd ratio of personal history of urolithiasis (0.16, p:0.00), salty food intake (0.39, p:0.02), family history of urolithiasis (0.39, p:0.01), and hypertension (1.77, p:0.04) was significantly associated with a greater risk of urolithiasis.

**Conclusion:**

The prevalence of urolithiasis in this study population is 4.04%. It affects males and females equally; 61.4% are in the age group of 25–64 years. Hypertension, high salt diet, personal history of urolithiasis and family history of urolithiasis are significant risk factors.

## 1 INTRODUCTION

Urolithiasis is a prevalent disease in Malaysia. The real prevalence of urolithiasis is not well studied in Malaysia. Worldwide, the prevalence ranges from 7% to 13% in North America, 5% to 9% in Europe and 1% to 5% in Asia.^1^, ^2^ The highest prevalence reported is 20% in Saudi Arabia.^3^ The prevalence varies widely in different regions and depends greatly on geographic area, racial distribution, socio‐economic status and dietary habits. The actual prevalence of urolithiasis may be higher than the estimation from hospital data. Thirty percent to 80% of stones will pass spontaneously, and these patients usually do not require medical attention.^4^ The increase in the incidence of urolithiasis in both the developed and the developing countries during the past decades has contributed to the rise of the healthcare burden.^4^

## 2 OBJECTIVE

In Malaysia, there have not been many studies conducted on the incidence and prevalence of urolithiasis. The pioneer study of urolithiasis in Malaysia is by Sreenevasan et al., who conducted a five yearly study on the incidence of urolithiasis in various tertiary hospitals in Peninsula Malaysia between the period of 1962 and 1981.^5^

A prevalence study which can represent the population should be conducted to provide a reliable and up‐to‐date information on urolithiasis. Thus, this study was undertaken to identify the prevalence of urolithiasis in population attending the primary healthcare centres in main cities of Sarawak. Demographic pattern and the associated factors of urolithiasis in this region were also studied.

The study was also undertaken to provide a platform for more studies to be conducted in the future in order to reduce the morbidity and mortality related to urolithiasis.

## 3 METHODS

### 3.1 Subjects

A cluster sampling method was used to select a cohort of participants aged ≥18 years old in Sarawak, Malaysia. Sarawak is the largest state among the 13 states in Malaysia, located in Northwest Borneo Island with a population of 2 636 000. Major health care services are provided in these four main cities of Sarawak (Kuching, Sibu, Bintulu and Miri). Five main primary healthcare clinics were chosen to represent the Sarawak populations according to the population to ethnicity ratio. Three clinics were then selected for the study using simple random sampling. Participants aged ≥18 years old and staying in the respective cities >6 months were conveniently selected from each clinic during the visit day. Participants who aged <18 years old, not willing to give consent and pregnant were excluded from the study. The allocation of percentage to each clinic was based on the total number of patients attending the respective clinic monthly as illustrated in Appendix S1, Figure 1.

Sample size of 1100 with a 20% drop out was calculated based on sample size calculation guidelines for logistic regression from observational studies for large population.^6^

### 3.2 Data collection

All data were collected by our research group investigators in the selected primary health care clinics. It was a single visit study where the participants were patients who attended the clinic on that particular day. A total of 70 visits were conducted throughout the research. Study method was illustrated as per Appendix S1, Figure 2. Each participant was informed regarding the study method and data collection. Informed consent was taken from the participants in the clinic prior to the recruitment into the study.

After informed consent was taken, each participant was required to undergo face‐to‐face interviews using a predesigned and standardised questionnaire. Information on demographic data including age, gender and race; diet and lifestyle; comorbidities, such as hypertension, diabetes mellitus and ischaemic heart disease; and personal and family history of urolithiasis was recorded. Diet variables that included consumption of water, source of water, subjective assessment of caffeine, salty food and red meat intake were recorded.

Age is categorised according to the Index Mundi Malaysia Age Structure as shown in Table 2.^7^

A ‘smoker’ is defined as smoking ≥100 cigarettes in one's lifetime. Exercise is defined as conducting physical activity >30 min per day, then categorised into frequent or occasional depending on the number of exercises conducted per week. Hypertension, diabetes mellitus and dyslipidaemia are defined as self‐reported premorbidity.

Ultrasonography of the kidney, ureter and bladder was performed by two trained investigators on all participants using Philip Lumify US machine, with a 3.0–5.0 MHz frequency probe, throughout this study. Details on presence of urolithiasis were recorded. Urine samples were analysed using urine dipstick in credentialed lab. Blood samples for analysis of creatinine, urea, calcium, uric acid and eGFR were taken and analysed.

## 4 ETHICAL CONSIDERATION

The study was performed according to the Declaration of Helsinki. This study was approved by the Malaysia Research and Ethics Committee of the Ministry of Health (NMRR‐18‐2588‐43 779 (IIR) dated 6 December 2018 until 3 February 2021.

## 5 STATISTICAL ANALYSIS

The prevalence of urolithiasis in Sarawak population was estimated to be ~4.0% according regional data.^4^, ^8^, ^9^ Sample size for prevalence was calculated using the Kish Leslie formula (1965), *n* = (Z_1−α_)^2^(P(1‐P)/D^2^) with an *α* of 0.05, a margin of error (D) of 2% and a sampling error of 10%. Sample size calculations suggested that 369 people would be needed in each site, with a predicted 10% refusal rate and then a total of 1107 peoples are required for three survey sites.

Prevalence of urolithiasis was studied, and relevant characteristics were described and stratified according the presence of urolithiasis. Personal history of urolithiasis, ultrasonographic proven urolithiasis and the life time prevalence was calculated overall and by age group, gender, body mass index (BMI) and family history of urolithiasis. Pearson Chi‐square test was used to compare the differences between categorical variables. The comparison between continuous variables was carried out using *T*‐test. The variables include age, gender, smoking, personal history of urolithiasis, family history of urolithiasis, BMI, fluid intake, occupation, caffeine intake, salty food intake, red meat intake, comorbidities such as hypertension, diabetes, ischemic heart disease, race and blood investigation such as eGFR, calcium (hypercalcaemia, Ca > 2.6 mmol/L), uric acid (hyperuricemia, Uric acid > 416 μmol/L) and urine PH level. All *p* values were two‐tailed, and findings at *p* < 0.05 were considered statistically significant. The statistical analyses were performed using the Statistical Package for the Social Sciences software (SPSS, version 24.0, IBM, Armonk, USA).

## 6 RESULTS

A total of 1100 participants were screened during the clinic visits, and 1087 participants completed the questionnaire, ultrasonographic examination and lab investigation with an overall response rate of 98.8%. Among the 1087 subjects, 486 (44.7%) were male and 601 (55.3%) were female. The mean age of the participants in this study was 50.24 (SD 14.3, range 17–89).

Among these 1087 participants in this study, a total of 44 participants (22 male and 22 female) have urolithiasis based on the presence of stone in the genitourinary tract scan at the time of the study. Prevalence of ultrasonographic proven urolithiasis calculated based on this cohort was 4.04%. Crude prevalence distribution of urolithiasis by demography is depicted in Table 1. The prevalence of urolithiasis based on self‐reported personal history of urolithiasis (those who has a history of urolithiasis) in the questionnaire was 2.94% (*n* = 32). Consequently, the overall lifetime prevalence of urolithiasis was 6.43% which include participants who had a history of kidney stones by self‐report and ultrasound proven urolithiasis.

**TABLE 1:** Crude prevalence distribution of urolithiasis by demography

	Study cohort, *n*	Ultrasound confirmed prevalence		
		*N*	%	*P* value
Total	1087	44	4.04	
Gender				0.471
Male	486	22	4.5	
Female	601	22	3.7	
Age				0.646
18–24	63	2	3.2	
25–54	595	27	4.5	
55–64	254	8	3.1	
>65	175	7	4.0	
BMI				0.725
<18.5	39	2	5.1	
18.5–22.9	199	8	4.0	
23–24.9	178	6	3.4	
>25	671	28	4.1	
Family history				
Yes	92	8	8.7	0.018
No	995	36	3.6	

Among the participants with urolithiasis, 13.6% of them have personal history of urolithiasis and 18.2% of them have family history of urolithiasis. The mean age of participants with urolithiasis was 50.05 (SD 14.6), whereas the most common age group for urolithiasis was 25–64 years old which accounts for 79% of the prevalence (Table 2). In terms of gender, both men and women have almost an equal prevalence of urolithiasis with a ratio of (M:1.2; F:1) in this study. Three main races with the highest prevalence of urolithiasis were Malay (45.5%), Iban (27.3%) and Chinese (13.6%), followed by other races (9.1%) and Bidayuh (4.5%).

**TABLE 2:** Frequency of urolithiasis according to age group

Age group	Frequency	Percentage (%)
18–24 (early working age)	2	4.5
25–54 (prime working age)	27	61.4
55–64 (mature working age)	8	18.2
>64 (elderly)	7	15.9
Total	44	100

Univariate analysis showed that odd ratios of personal history of urolithiasis (0.16, *p*: 0.00), salty food (0.39, *p*: 0.02), family history of urolithiasis (0.39, *p*: 0.01) and hypertension (1.77, *p*: 0.04) were significantly associated with a greater risk of urolithiasis. The odd ratio of getting urolithiasis was depicted in Table 3. The size and location of the urolithiasis are shown in Table 4. Kidney stones with a diameter of less than 10 mm were the most common in our study.

**TABLE 3:** Univariate‐adjusted odd ratio for urolithiasis among the cohort aged >18 years

Variable	Subjects		*p* value	Odd ratio (95% CI)
	No urolithiasis	Urolithiasis present		
Personal history of urolithiasis			0	0.16(0.06, 0.42)
Yes	26 (81.2%)	6 (18.8%)		
No	1017 (96.4%)	38 (3.6%)		
Family history of urolithiasis			0.018	0.39 (0.18, 0.88)
Yes	84 (91.3%)	8 (8.7%)		
No	959 (96.4%)	36 (3.6%)		
Hypertension			0.077	1.77(0.93, 3.39)
Yes	570 (95.0%)	30 (5.0%)		
No	473 (97.1%)	14 (2.9%)		
Prefer salty food			0.002	0.39 (0.21, 0.73)
Yes	402 (93.7%)	27 (6.3%)		
No	641 (93.3%)	17 (6.7%)		

**TABLE 4:** Characteristic of urolithiasis in the cohort

Characteristic	*N* (%)
Location	
Kidney	39 (88.6)
Ureter	4 (9.1)
Bladder	1 (2.3)
Stone size (mm)	
<10	35 (79.6)
10–19	6 (13.6)
>20	3 (6.8)

Our study showed that hypercalcemia was found in seven participants (0.6%), but none of them had urolithiasis. Hyperuricemia was found in 320 participants (29.4%), 307 in nonurolithiasis cohort and 13 in urolithiasis cohort. There was no significant difference between urolithiasis and nonurolithiasis in the blood analysis. Estimated glomerular filtration rate (GFR) was calculated based in the NKF classification of Chronic Kidney Disease (CKD), and CKD stages was not a significant risk factor for urolithiasis.

## 7 DISCUSSION

Urolithiasis is defined as the presence of stones in genitourinary tract. The prevalence of urolithiasis in Malaysia is yet to be determined with a proper community‐based epidemiological study. It is a need to understand that the natural history of progressive stone disease is urosepsis and mortality. Therefore, it is important that preventive measures be undertaken instead of curative for urolithiasis. To undertake these preventive measures, a proper epidemiological study has to be conducted to know the demographic pattern and the associated factors.

A community‐based study is required to get the actual data on epidemiological data on urolithiasis in the population. It requires support from multiple sectors of healthcare providers, funds, effort and time. To our knowledge, most of the community‐based epidemiological studies concerning urolithiasis was done based on questionnaires and retrospectively extracted hospital data. It is difficult to determine the prevalence of urolithiasis in some region in view of the limited availability of data and sources of documentation for urolithiasis especially in Sarawak, Malaysia. However, community‐based studies using questionnaires raise the possibility of overestimation and/or underestimation of the actual disease prevalence. This problem explains in part the diversity of the reported prevalence data in Asian country (Table 5). Therefore, we conducted an opportunity study on ultrasonographic proven prevalence of urolithiasis in Sarawak, Malaysia.

**TABLE 5:** Studies conducted in Asia

Country/nation/region/city	Reference	Prevalence
China	Zeng Q, He Y^9^	4.0%
China	Luo^10^	1‐5%
Taiwan	Lee et al^11^	9.6%
South Korea	Kim et al^4^	3.5%
Japan	Ogawa^8^	4%
Saudi Arabia	Robertson WG^3^	20%
Malaysia	Current study	4%

The prevalence reported in our study is 4% which is in line with other studies that were conducted in East Asian countries such as Japan (4%), China (4%) and Korea (3.5%).^4^, ^8^, ^9^, ^10^ Prevalence that was reported among the Asian countries is highly variable (1%–20%) as depicted in Table 5.

There is a significant evidence that systemic disorder increases the risk of urolithiasis including obesity, diabetes mellitus, hypertension, coronary heart disease and metabolic syndrome.^12^ In our study, hypertension was reported as a significant risk factor for urolithiasis. [Odd ratio: 1.77(CI 0.93, 3.39)]. It may be due to high salt intake in this region that can be predisposed to hypertension, leading to urolithiasis. This is in keeping with a study by Cappuccio et al. who reported that hypertensive men had a greater risk of developing urolithiasis than normotensive ones.^13^

Scales et al. also reported that urolithiasis is more common among obese than normal weight individual. He also stated that the increasing level of obesity may be a contributing factor to the rising rates of stone disease in the United States.^14^ Nowfar et al. also reported that a significant positive correlation exists between obesity and urolithiasis for both genders.^15^ In our study, 61.73% individuals have a BMI of >25. Eric N Taylor et.al. reported that a history of diabetes mellitus was independently associated with a history of nephrolithiasis. People with Type 2 diabetes mellitus have highly acidic urine that can lead to kidney stones, particularly uric acid stones.^16^ However, there is no significant correlation between BMI and diabetes to prevalence of urolithiasis in our study. This could be due to the opportunistic nature of the study that conducted in a primary health care clinic instead of a population study.

Urolithiasis develops more frequently in individuals with a family history of urolithiasis than in those with no family history as specified in our study [Odd ratio: 0.39 (CI 0.18, 0.88)]. This result is in congruent with a study by Nalini et al. in India that showed that there is a significant association between family history and urolithiasis. Nalini et al. proved that a family history of urolithiasis has been reported in 16% to 37% of patients who have formed a kidney stone, compared with 4% to 22% in healthy control subjects.^17^

As mentioned above, family history played an important role in contributing to urolithiasis, suggesting that genetic or family‐related environmental factors were also important in urolithiasis formation.

Diet plays an important role in the development of urolithiasis especially in patients who are predisposed to this condition. This is consistent with our study showing a higher prevalence of urolithiasis in high salt diet intake participants [Odd ratio: 0.39 (CI 0.21, 0.73)]. This is because of an excess sodium intake predispose to hypercalciuria, and this may increase the risk of urolithiasis. This is supported by a report from Women's Health Initiative Observational Study by Sorenson et al. which showed that higher dietary sodium intake increased the risk of urolithiasis formation by 11% to 61%.^18^

A diet rich in animal protein is believed to increase the risk of urolithiasis as the excess of animal protein intake is thought to cause transient urinary acidification with subsequent decreased urinary citrate and the increased of undissociated uric acid excretion. However, in our study, preference of red meat intake did not appear to increase the risk of urolithiasis. This finding is consistent with the report from Women's Health Initiative Observational Study by Sorenson et al. that showed dietary animal protein intake was not independently associated with incident urolithiasis on multivariate analysis.^18^

It is difficult to perform a community‐based study involving whole age groups, and it is important to carefully select the age group, because the prevalent age group is different according to the nature of the disease. The prevalence of urolithiasis varies by age with a low prevalence in children and the elderly. However, the rates are peaking in the fourth to sixth decades of life.^19^ Therefore, our study was only conducted on participants aged ≥18 years old. The peak age of highest prevalence of urolithiasis in our study is 25–64 years old (79.6%, *N*: 35). This is consistent with a nationwide study which was conducted in Japan showing that the peak age of the first episode of upper urinary tract stones is in middle age was from 30 to 60 years old.^8^ The high prevalence of urolithiasis in the middle age group may be related to diet, work and lifestyle changes as our lifestyle and dietary habits in Asia have also become increasingly westernised over the past 60 years.

Throughout the century, a persistent male predominance has been found in the prevalence and incidence of urolithiasis which contradict our current study. In Malaysia, a study that was reported by Sreenevasan et al. in 1980 also showed an overall gender ratio of three males to one female.^5^ Our study showed almost an equal prevalence in male and female with a ratio of 1.2:1. In the recent years, there has been some evidence on gender gap narrowing which may be related to the change in dietary habit and the increase in the incidence of metabolic syndrome.^20^ In the United States, the report showed that although males were more likely to be affected by urolithiasis than females, there has been a decrease from 1.7 to 1.3 based on the nationwide inpatient sample that was carried out from 1997 to 2002.^20^

Rising of global temperatures could lead to an increase in urolithiasis, according to a research presented at the 103rd Annual Scientific Meeting of the American Urological Association (AUA). Robertson et al. stated that there is an increase in the incidence of urolithiasis in the tropics where the risk of stone formation is compounded by low urine volume.^21^ However, there is no significant correlation between fluid intake to urolithiasis formation in our study. This could be due to the reason that our data are a subjective assessment through questionnaire instead of a quantitative measure.

The strength of our study is that this is the first prospective study that was conducted in Malaysia on the prevalence of ultrasonographic proven urolithiasis. The ultrasound study was conducted only by two investigators, thereby reducing any inter observer discrepancy on ultrasound findings and data collection methods. The response rate was excellent, at 98%, as it was conducted as a single visit study for participant's convenience and compliance.

### 7.1 Limitations

The limitation of our study includes the fact that it is a cross‐sectional design, and it may limit the causal inference for risk factors. Secondly, ultrasonographic proven urolithiasis might lead to exclusion of small stones and leading to an underestimation of stone prevalence.

## 8 CONCLUSIONS

The prevalence of urolithiasis in this study population of Sarawak is 4.04%. It affects males and females equally; the commonest age group is 25–64 years. Hypertension, high salt diet, personal history of urolithiasis and family history of urolithiasis are significant risk factors. In the prevention of urinary stone disease, measures must be taken to reduce these. Nevertheless, our study provides important information for Malaysia's epidemiological data on urolithiasis.

## ETHICAL APPROVAL AND CONSENT TO PARTICIPATE

Ministry of Health and Medical Research Ethics Committee has reviewed this study's protocol code (NMRR‐18‐2588‐43 779) and granted ethical approval and consent from 6 December 2018 to 3 February 2021. Signed consents were taken from the participants.

## CONSENT FOR PUBLICATION

Written consent for publication was obtained from each participant.

## CONFLICT OF INTEREST

The authors declare that they have no competing interests.

## AUTHOR CONTRIBUTION

Dr. Kamal Raj, the first author, made significant contributions to the research design, data collection and interpretation and writing of the manuscript. Dr. Richelle Chua Huey Bing also helped in the manuscript writing and was involved in the research design and the collection of the data. Dr. Teh Guan Chou and Dr. Clarence Lei Chang Moh who have expertise in quantitative research were involved in the research design and interpretation of the data and contributed important intellectual input in manuscript writing. All the authors have critically reviewed and approved the final draft and are responsible for the content of the manuscript.

## Supporting information


**Appendix S1.** Figure 1: Flowchart of sampling method
Figure 2: Flowchart on study method

## Data Availability

Data generated and analysed during the current study are not publicly available as individual privacy could be comprised; however, it may be available from the corresponding author on reasonable request.
