# Supplementary material for: Prevalence of urolithiasis in Sarawak and associated risk factors: An ultrasonagraphy‐based cross‐sectional study
Source: BJUI Compass. 2022 Apr 20;4(1):74–80. doi: 10.1002/bco2.152 (PMC9766857; doi:10.1002/bco2.152)
Supplement: Supplementary file 1 — Appendix S1. Figure 1: Flowchart of sampling method Figure 2: Flowchart on study method [file BCO2-4-74-s001.docx]

**Appendix S1**

**Figure 1: Flowchart of sampling method**

**Figure 2: Flowchart on study method**

**Questionnaire**

**Sarawak Stone Project Performa ( English version)**

1. NRIC :
2. Age :
3. Sex: Male / Female
4. Smoker / Non smoker
5. Personal history of renal stone : Yes / No
   - If Yes
     1. Treatment method and duration
     2. History of passing out stone without any treatment
6. Any family history of kidney stone : Yes / No
7. BMI :
   - Height : ………m
   - Weight : ……… kg
   - BMI:
8. Fluid Intake per Day
   - < 500mls
   - 500-1500mls
   - 1500- 2500mls
   - >2500mls
9. Occupation
   - Indoor
   - Outdoor
   - No job
10. Source of water
    - Tap water
    - Mineral water
    - River / Mountain water
11. Amount of exercises
    - None
    - Frequent > 3 times/ week
    - Occasional < 3 times/ week
12. Caffein Intake ( Coffee / Tea)
    - Never
    - Occasional > 3 times/ week
    - Frequent < 3 times/ week
13. Salty Food
    - Prefer
    - Not Prefer
14. Red meat Intake
    - Prefer
    - Not Prefer
15. Co-morbidity
    - Hypertension
    - Diabetes
    - Coronary Heart Disease
16. Races
    - Chinese
    - Iban
    - Bidayuh
    - Malay
    - Indian
    - Others

Blood Test

| Urea |  |
| --- | --- |
| Creatinine |  |
| Calcium |  |
| Uric Acid |  |
| eGFR |  |

UFEME - pH

Ultrasound Findings

Right kidney

- Stone
- No stone
- Hydronephrosis
- No hydronephrosis

Left kidney

- Stone
- No stone
- Hydronephrosis
- No hydronephrosis

Bladder

- Stone
- No stone
